# Enhancing fine‐tuning efficiency and design optimization of an eight‐channel 3T transmit array via equivalent circuit modeling and Eigenmode analysis

**DOI:** 10.1002/mp.17612

**Published:** 2025-01-15

**Authors:** Ehsan Kazemivalipour, Ergin Atalar

**Affiliations:** ^1^ Department of Electrical and Electronics Engineering Bilkent University Ankara Turkey; ^2^ National Magnetic Resonance Research Center (UMRAM) Bilkent University Ankara Turkey; ^3^ A. A. Martinos Center for Biomedical Imaging Department of Radiology Massachusetts General Hospital Charlestown Massachusetts USA; ^4^ Harvard Medical School Boston Massachusetts USA

**Keywords:** coil tuning, co‐simulation, eigenmode analysis, equivalent circuit modeling, fine‐tuning process, transmit array

## Abstract

**Background:**

Radiofrequency (RF) transmit arrays play a crucial role in various MRI applications, offering enhanced field control and improved imaging capabilities. Designing and optimizing these arrays, particularly in high‐field MRI settings, poses challenges related to coupling, resonance, and construction imperfections. Numerical electromagnetic simulation methods effectively aid in the initial design, but discrepancies between simulated and fabricated arrays often necessitate fine‐tuning. Fine‐tuning involves iteratively adjusting the array's lumped elements, a complex and time‐consuming process that demands expertise and substantial experience. This process is particularly required for high‐Q‐factor arrays or those with decoupling circuitries, where the impact of construction variations and coupling between elements is more pronounced. In this context, our study introduces and validates an accelerated fine‐tuning approach custom RF transmit arrays, leveraging the arrays equivalent circuit modeling and eigenmode analysis of the scattering (S) parameters.

**Purpose:**

This study aims to streamline the fine‐tuning process of lab‐fabricated RF transmit arrays, specifically targeting an eight‐channel degenerate birdcage coil designed for 3T MRI. The objective is to minimize the array's modal reflected power values and address challenges related to coupling and resonance.

**Methods:**

An eight‐channel 3T transmit array is designed and simulated, optimizing capacitor values via the co‐simulation strategy and eigenmode analysis. The resulting values are used in constructing a prototype. Experimental measurements of the fabricated coil's **S**‐parameters and fitting them into an equivalent circuit model, enabling estimation of self/mutual‐inductances and self/mutual‐resistances of the fabricated coil. Capacitor adjustments in the equivalent circuit model minimize mismatches between experimental and simulated results.

**Results:**

The simulated eight‐channel array, optimized for minimal normalized reflected power, exhibits excellent tuning and matching and an acceptable level of decoupling (|*S_nn_* |≤‐23 dB and |*S_mn_* |≤‐11 dB). However, the fabricated array displays deviations, including resonances at different frequencies and increased reflections. The proposed fine‐tuning approach yields an updated set of capacitor values, improving resonance frequencies and reducing reflections. The fine‐tuned array demonstrates comparable performance to the simulation (|*S_nn_* |≤‐15 dB and |*S_mn_* |≤‐9 dB), mitigating disparities caused by construction imperfections. The maximum error between the calculated and measured **S**‐parameters is −7 dB.

**Conclusion:**

This accelerated fine‐tuning approach, integrating equivalent circuit modeling and eigenmode analysis, effectively optimizes the performance of fabricated transmit arrays. Demonstrated through the design and refinement of an eight‐channel array, the method addresses construction‐related disparities, showcasing its potential to enhance overall array performance. The approach holds promise for streamlining the design and optimization of complex RF coil systems, particularly for high Q‐factor arrays and/or arrays with decoupling circuitry.

## 1 INTRODUCTION

Radiofrequency (RF) transmit (Tx) array coils, utilizing multiple parallel transmitting elements to offer additional degrees of freedom (DoF), have been extensively investigated for various applications. These applications encompass enhancing field uniformity,^1^, ^2^, ^3^ reducing RF pulse duration,^4^ enabling RF shimming while mitigating SAR hotspots,^5^, ^6^, ^7^ improving power efficiency,^8^, ^9^ facilitating catheter tracking,^10^ and executing implant‐friendly modes.^11^, ^12^, ^13^, ^14^, ^15^, ^16^ The performance of Tx arrays can significantly benefit from parallel transmission technology when the coil designs meet particular specifications, such as minimizing coupling among individual array elements and ensuring appropriate interaction with subjects under test to achieve sufficient B_1_^+^ field efficiency. High coupling levels present a significant design challenge, resulting in extra reflected power from the coil and a decrease in the power delivered to the subject.^8^ To address this challenge, various approaches are employed to reduce effective coil coupling, including the use of L/C decoupling networks,^17^, ^18^ overlapping neighboring array elements,^19^, ^20^ transformer decoupling,^21^ inserting induced current elimination (ICE) decoupling elements,^22^, ^23^ and incorporating reactive decoupling circuits among adjacent array elements.^24^ Increasing the number of array elements enhances DoF for RF shimming and excitation encoding capabilities. However, higher coupling among individual array elements conversely decreases DOF as B_1_^+^ patterns become more similar. The challenge intensifies with an increasing number of array elements, requiring additional decoupling components and longer RF cables to address the coupling issue^8^, ^25^, ^26^ Due to these factors, designing Tx arrays is notably more challenging than conventional Tx coils.

Full‐wave electromagnetic (EM) models^27^, ^28^, ^29^, ^30^ have primarily been employed in simulation environments to design Tx arrays and study their interactions with subjects under test. Although accurate, full‐wave modeling approaches are time‐consuming.^31^ In the performance optimization of an array, relying solely on full‐wave simulations becomes a significant limiting factor, as each tuning condition necessitates a separate full‐wave simulation. To expedite full‐wave EM simulations, a co‐simulation strategy^32^ has been employed. This process replaces all lumped components with equivalent lumped ports and calculates coil multi‐port scattering (**S**) matrices through a full EM field simulation, later determining the optimal lumped elements in a short time using an RF optimizing tool. Recently, we developed an approach based on eigenmode analysis of the **S**‐matrix^33^ to categorize the excitation modes of a pre‐designed Tx array according to their level of power reflections. Eigenmode analysis can be employed during the optimization procedure to expand the excitation space of Tx arrays with a low level of total reflected power.

In a typical coil‐building process, once the fundamental structure of the coil is determined, the values of discrete elements (primarily capacitors) are computed through an optimization process, followed by the fabrication of the coil. In the case of lab‐fabricated arrays, especially those with high‐Q‐factors or those with decoupling circuitries, the discrete elements derived from full‐wave simulations may require adjustments to ensure that the measurement results are as optimal as the simulation results. Mismatches between measurement and simulation results can arise from several factors, including variations in the dimensions of the coil channels, deviations in their relative positioning, and misalignments with respect to the RF shield (if present). Even minor differences in the physical construction—such as variations in copper segment sizes and spacing—can significantly alter the coil's EM properties. Additionally, inconsistencies in the assembly process, such as shifts in the placement or orientation of the coil components, may lead to differences in resonance frequencies, coupling levels, and overall performance when compared to the simulated design. To overcome these differences, coil designers commonly initiate the process using optimized components derived from simulations supplemented by variable components. Fine‐tuning and matching procedures are then conducted to minimize disparities between simulation and experimental outcomes.^34^, ^35^ In this process, the array elements iteratively can be tuned and matched one by one in successive steps. This iterative process becomes increasingly intricate and time‐consuming as the number of Tx channels increases, requiring expertise and substantial experience.

In this study, we propose an accelerated fine‐tuning of Tx arrays through equivalent circuit modeling and eigenmode analysis. After constructing a Tx array based on EM simulation, we experimentally measure the **S**‐parameters of the fabricated coil and fit them into an equivalent circuit model. Adjustments to the capacitors integrated with the circuit model are suggested to optimize the performance of the fabricated coil and ensure that it achieves results comparable to those from simulations. Equivalent circuit modeling, previously used to find optimal lumped elements of simulated arrays,^36^, ^37^, ^38^, ^39^, ^40^, ^41^ is now applied in the fine‐tuning process of Tx arrays.

To showcase the effectiveness of our proposed fine‐tuning method, we simulate and fabricate an eight‐channel head Tx array for a 3T MRI system. The array prototype, designed in a birdcage‐like shape, inherently exhibits higher efficiency compared to loop‐based cylindrical array models due to shared conductors between neighboring meshes.^34^, ^42^ Furthermore, the design ensures that all resonating modes collapse to a single (degenerate) frequency.^34^, ^42^, ^43^ When operated in circularly polarized (CP) mode, this prototype performs nearly as well as conventional CP‐driven head birdcage coils.^44^, ^45^, ^46^, ^47^ Given the capacitive decoupling of neighboring channels in this array, the fine‐tuning and matching of capacitively decoupled coils are intricate, as adjustments to one coil's channel can impact the performance of other channels. Therefore, special attention is given to including the exact structure of the fabricated coil in the design process. We utilize the equivalent circuit model of the fabricated coil, considering all magnetic and electrical couplings,^19^, ^38^, ^39^ to determine all inductance and resistance values required for the circuit model. Subsequently, we employ eigenmode analysis of the **S**‐matrix to obtain the optimum capacitor values, optimizing them to minimize the array's total reflected power.

## 2 THEORY

**FIGURE 1:**
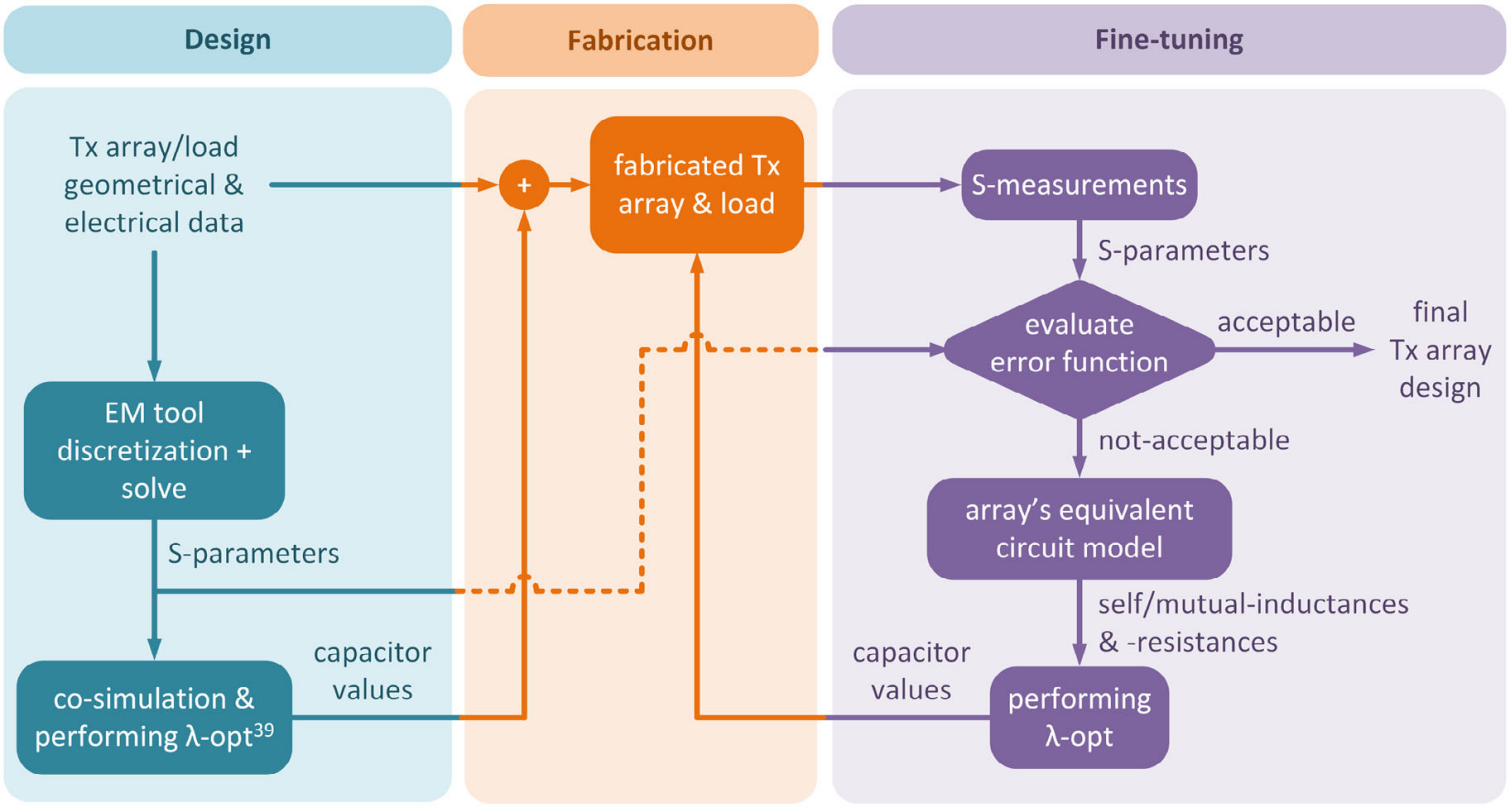
Workflow of the proposed fast fine‐tuning approach.

For lab‐fabricated Tx arrays, a mismatch might exist between the measured and simulated **S**‐parameters. To address this mismatch, we propose an iterative, practical, and rapidly deployable fine‐tuning process. This method is grounded in integrating the equivalent circuit model of the fabricated coil with the capacitance values optimized through simulating the coil structure. Figure 1 outlines the workflow of this proposed method. Initially, a commercial EM solver is employed to simulate the array's structure, determining optimal values for the coil's capacitors in the simulation environment. Subsequently, by measuring the **S**‐parameters of the fabricated coil across a wide frequency range and analyzing its equivalent circuit model, we can estimate the self/mutual‐inductances and ‐resistances of the array's circuit model. Once these parameters are determined, fine‐tuned capacitor values are obtained by minimizing the error function for the fabricated coil, as detailed in the subsequent section. The **S**‐parameters corresponding to the updated capacitor values are then measured. If the measured **S**‐parameters fail to meet the predefined design criteria, the process of iteratively refining inductances and resistances is repeated until the criteria are fulfilled.

## 3 METHODS

### 3.1 Array design and simulation

**FIGURE 2:**
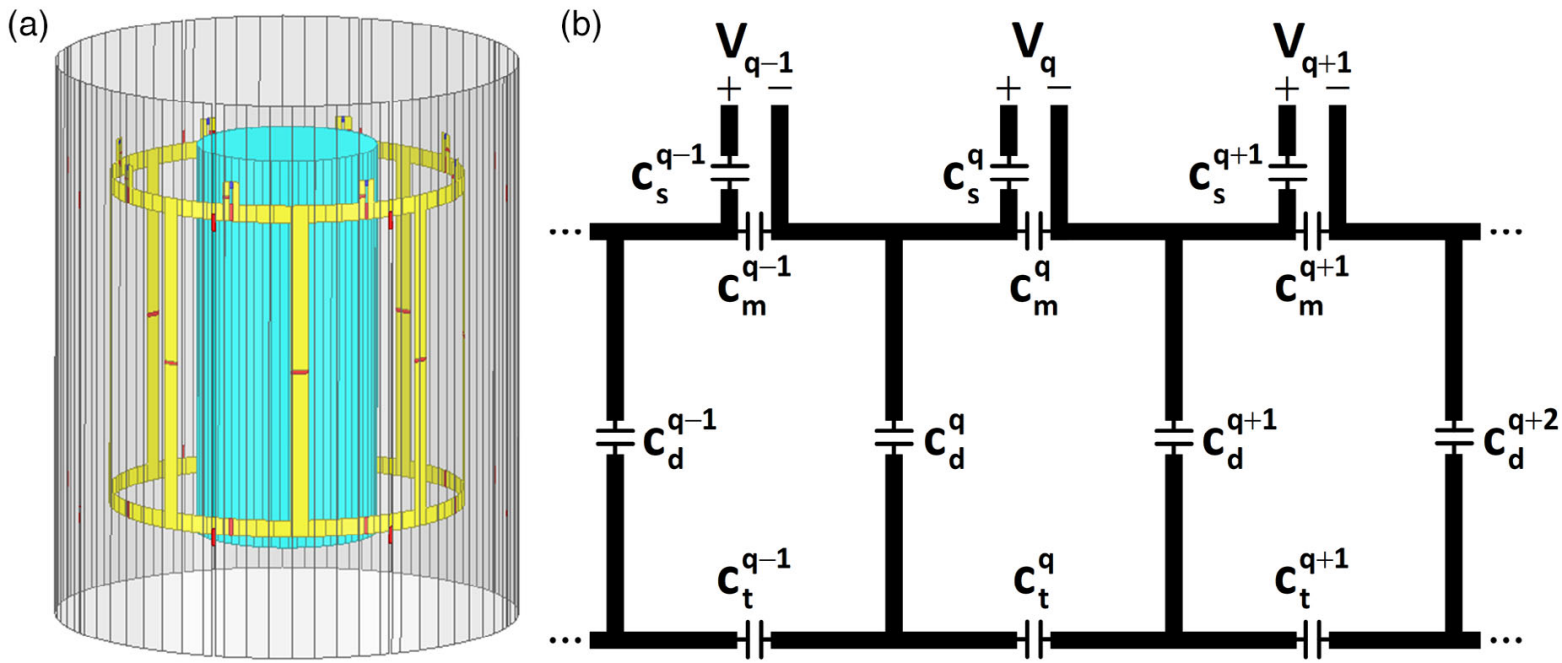
(a) EM simulation model and (b) schematic of a loaded 8‐channel degenerate birdcage array. *V_q_* for *q = 1, 2, …, 8* denotes the voltage across the port of the *q* ^th^ loop. Decoupling capacitors are labeled as $c_{d}^{q}$, and tuning capacitors, matching capacitors, and series matching capacitors are denoted by $c_{t}^{q}$, $c_{m}^{q}$, and $c_{s}^{q}$, respectively.

Figure 2 displays the model and schematic of the simulated eight‐channel degenerate birdcage Tx array. The coil has a cylindrical shape with a diameter of 315 mm and a length of 300 mm. The width of all end‐rings and rungs is 15 mm. The coil is shielded with a cylinder having a diameter of 412 mm and a length of 500 mm. To mitigate eddy currents induced by gradient fields,^48^ the shield is segmented into eight evenly distributed sections along the axial direction. The adjacent slits are connected via two 3 nF capacitors positioned facing the coil's end‐rings. A cylindrical phantom (diameter: 153 mm, length: 350 mm, conductivity: 0.62 S/m, and relative permittivity: 80) is placed in the coil center.

The array's adjacent transmit channels were decoupled by adjusting the capacitors between the neighbors, as depicted in Figure 2b and denoted by $c_{d}^{q}$. In this figure, $c_{t}^{q}$, $c_{m}^{q}$, and $c_{s}^{q}$ represent tuning capacitors, matching capacitors, and series matching capacitors, respectively.

Numerical simulations were conducted using ANSYS Electronics Desktop (ANSYS Inc., Canonsburg, Pennsylvania, USA). All coil and shield conductors were assigned properties equivalent to copper, utilizing a finite conductivity boundary.^49^ To ensure the precision of our simulation, we carefully regulated the mesh sizes. Initially, we maintained them at values below 2 mm for array conductors, less than 10 mm in the shield, less than 1 mm for lumped ports and capacitors, and under 10 mm within the phantom. ANSYS HFSS employs an iterative adaptive meshing strategy, continuously refining initial meshes in each iteration while computing and monitoring **S**‐parameters. The convergence criterion is met when the maximum change in **S**‐parameter magnitudes between consecutive iterations is below the predetermined threshold of 0.01. The co‐simulation strategy,^32^ which combines the finite element method and circuit simulation analysis, was employed to optimize capacitor values. We extensively utilized this method in our prior studies.^13^, ^33^, ^46^, ^47^, ^50^, ^51^ Since the geometry of the simulated array was circularly symmetric, capacitor values within the same sections of different channels were assumed to be equal. Four lossy capacitors $\left(\bar{c}=\left[\begin{array}{cccc} c_{d} & c_{t} & c_{m} & c_{s} \end{array}\right]\right)$ were treated as free design parameters, with capacitor losses modeled by introducing resistances in series. These resistances were estimated through linear interpolation based on a discrete set of series resistances extracted from standard capacitor datasheets.

Eigenmode analysis of the **S**‐matrix^33^ was employed for the effective design of the array in terms of power efficiency. The optimization objective was to minimize the normalized reflected power for all excitation eigenmodes (eigenvalues of **S^H^S**, i.e., *λ_n_* values, see Appendix A for more detailed description) and the CP excitation mode (*λ_CP_*) at 123.2 MHz. The optimization problem was formulated as follows to find the capacitor values (λ‐opt):

(1) $$\min_{\bar{c}}\frac{1}{8}\sum_{n=1}^{8}\lambda_{n}^{2}+\lambda_{CP}^{2}$$

Minimizing the sum of squared eigenvalues inherently provides more weight to larger eigenvalues due to the squaring operation, leading higher eigenvalues contribute disproportionately to the total, ensuring that significant eigenvalues are reduced more effectively than with a simple average. It is noteworthy that the CP excitation mode is critical for producing a uniform B_1_^+^ field in a large volume for the 3T degenerate birdcage coil. In this mode, all input ports were individually excited with the same power and a phase shift of 45° between successive ports. Including *λ_CP_* in the minimization problem results in those capacitor values that enable the CP excitation mode of the resulting array to be composed of the most efficient excitation eigenmodes.^33^

### 3.2 Array construction and prototype

**FIGURE 3:**
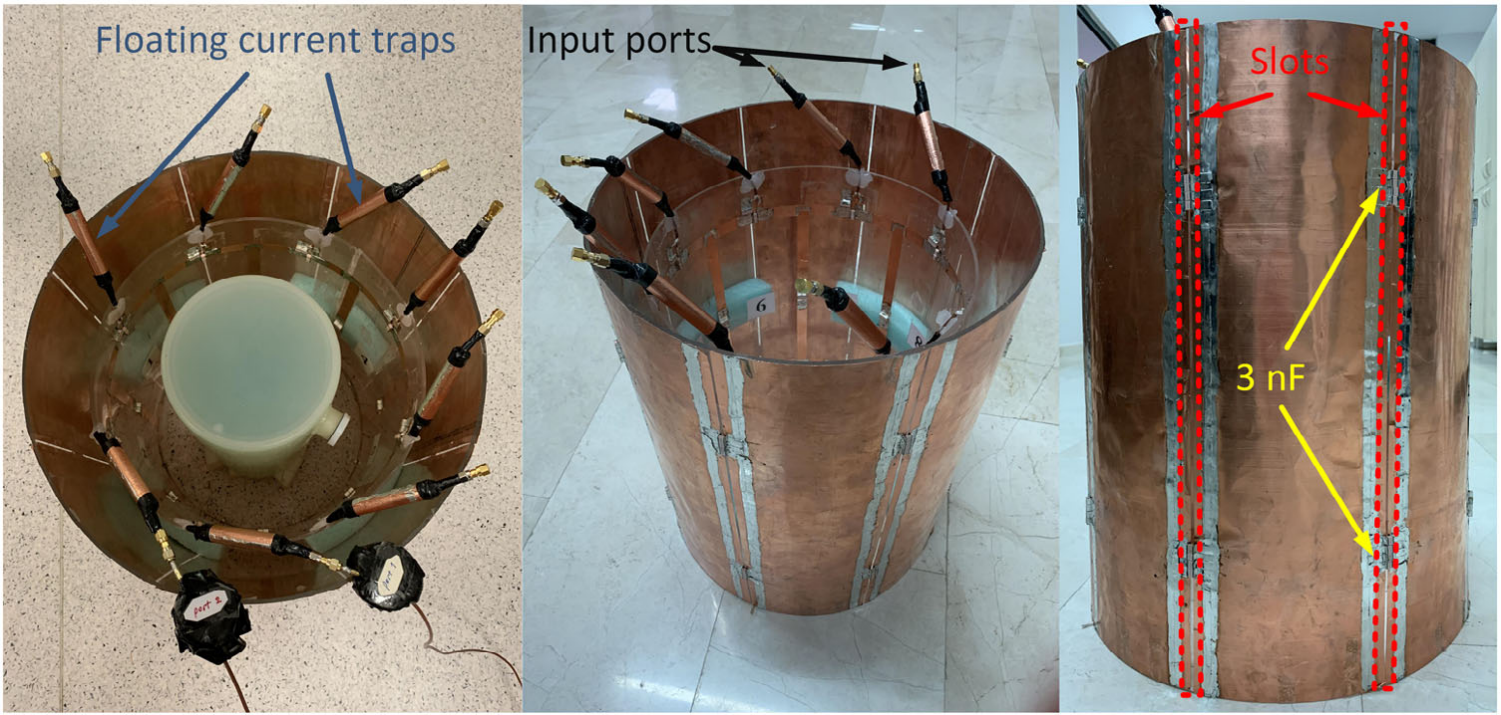
Fabricated structure of an eight‐channel degenerate birdcage array loaded with a cylindrical sodium‐nickel solution phantom. The shield comprises eight equally spaced segments, connected by two 3‐nF capacitors between adjacent segments at positions facing the coil's end‐rings. Additionally, eight floating current traps are integrated into the coil structure.

Figure 3 depicts the constructed array, with dimensions corresponding to the simulated coil shown in Figure 2a. Two cylindrical plexiglasses support the coil and shield structures. The shield is broken down into eight uniformly spaced segments distributed along the azimuthal direction. All coil and shield segements consist of copper strips with a thickness of 35 µm. To mitigate common‐mode currents at 123.2 MHz, eight floating current traps were integrated into the coil structure.^52^, ^53^ The optimized capacitors achieved from the simulation were implemented in the coil's prototype. The adjacent shield segments were connected with two 3 nF capacitors located facing the coil's end‐rings. All capacitors utilized were non‐magnetic surface mount high‐Q capacitors. A phantom (USA Instruments Inc., containing 3.7 g/L NiCl_2_.6H_2_O and 2.4 g/L NaCl) with a cylindrical shape and a diameter of 153 mm was used for loading. The phantom's conductivity, measured using the magnetic resonance electrical properties tomography (MREPT) technique,^54^ was determined to be 0.62 S/m. The relative permittivity of the phantom was assumed to be the same as the water relative permittivity of water.^55^, ^56^

### 3.3 Fabricated coil equivalent circuit model

**FIGURE 4:**
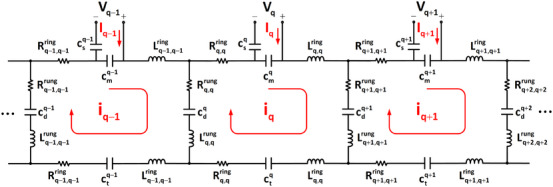
Equivalent lumped element circuit model of the fabricated eight‐channel degenerate birdcage array. The model includes self‐inductances and resistances, with mutual inductances and resistances considered but not explicitly shown.

Figure 4 presents the equivalent circuit model of the fabricated coil, encompassing the effects of all self/mutual‐inductances and self/mutual‐resistances originating from the coil, the shield, and the load. Eddy currents induced on the subject due to the current on a coil element result in an electromotive force on other coil elements, modeled as mutual resistance.^57^

In Figure 4, $L_{q,q}^{ring}$ and $R_{q,q}^{ring}$ denote the self‐inductance and ‐resistance of the *q* ^th^ arc at top/bottom end‐ring, while $L_{q,q}^{rung}$ and $R_{q,q}^{rung}$ denote the self‐inductance and ‐resistance of the *q* ^th^ rung. Although it is not shown in Figure 4, the mutual‐inductance and ‐resistance between the *q* ^th^ and *p* ^th^ arcs in the same end‐ring $\left(L_{q,p}^{ring}\;and\;R_{q,p}^{ring}\right)$ are also considered in the formulation, as well as mutual‐inductance and ‐resistance between the *q* ^th^ and *p* ^th^ arcs in different end‐rings $\left(L_{q,p}^{*ring}\;and\;R_{q,p}^{*ring}\right)$. Additionally, mutual‐inductance and mutual‐resistance between the *q* ^th^ and *p* ^th^ rungs $\left(L_{q,p}^{rung}\;and\;R_{q,p}^{rung}\right)$ are formulated.

According to the image theory, EM fields produced by currents induced on the RF shield's surface are equivalent to those produced by the image of the original currents running on the array. Thus, the RF shield effect can be modeled with an imaginary array with the same physical shape and radius of $r_{i}=r_{s}^{2}/r_{c}$, where $r_{c}$ and $r_{s}$ are the array and shield radii, respectively.^58^, ^59^ The imaginary currents have an opposite direction to the original currents to satisfy the boundary condition on the RF shield's surface. Consequently, the shield's effects are considered by adding a mutual‐inductance (‐resistance) between the *q* ^th^ arc and the image of the *p* ^th^ arc in the same end‐ring, denoted as 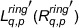. Similarly, the mutual‐inductance (‐resistance) between the *q* ^th^ arc and the image of the *p* ^th^ arc in different end‐rings are included in the circuit as ${L_{q,p}^{*\mathrm{ring}}}^{\prime }\left({R_{q,p}^{*\mathrm{ring}}}^{\prime }\right)$. Finally, the mutual‐inductance (‐resistance) between the *q* ^th^ rung and the image of the *p* ^th^ rung is labeled as 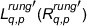.

Assuming zero current for the mesh formed by the lower end‐ring and analyzing the array's circuit model shown in Figure 4 with the Kirchhoff mesh current method, we can derive the coil's impedance matrix as a function of capacitors, inductances, resistances, and frequency (see Equation B10 and Appendix B for detailed derivation). In the equivalent circuit model of the fabricated coil, we estimate the inductance and resistance matrices by implementing the optimized capacitor values obtained from simulation to the fabricated coil and then measuring the **Z**‐parameters of the coil (refer to Equations B12 and B13 in Appendix B). With the inductance and resistance matrices determined at 123.2 MHz, we can adjust the capacitors to achieve the desired **S**‐parameters. The fine‐tuned capacitor values are identified by minimizing the error function (Equation 1) for the fabricated coil at 123.2 MHz. These optimized capacitors are then substituted for the initial ones in the fabricated coil, and the **S**‐parameters are remeasured. If the measured **S**‐parameters do not meet the predefined design criteria, additional iterations may be required to refine the inductance and resistance values until the criteria are satisfied.

### 3.4 Measurements

The **Z** and **S**‐parameters of the array were measured across a frequency range of 108.2–138.2 MHz using a calibrated Agilent E5061B vector network analyzer.

MRI experiment was conducted on a 3T scanner (Magnetom Trio A Tim, Siemens Healthcare, Erlangen, Germany) equipped with eight transmitters, each powered by an individual amplifier (Analogic Corp., Boston, Massachusetts, USA) capable of delivering up to 8 kW peak power. Eight coaxial cables, each fitted with bazooka baluns, transmitted the RF power from the amplifiers to the eight‐channel array. The Tx array functioned exclusively in Tx mode, without any detuning circuits. Signal reception was handled by a Siemens body‐matrix coil, a standard six‐channel flexible surface coil with integrated preamplifiers.

We acquired the B_1_^+^ map at the central axial plane of the coil using a method based on the Bloch‐Siegert shift technique.^60^, ^61^ This was implemented using a modified gradient‐echo (GRE) pulse sequence, applying an off‐resonance Fermi pulse to the spins. The pulse duration was 8 ms, with an off‐resonance frequency of 2 kHz. Key imaging parameters included TR/TE = 100 ms/12 ms, slice thickness = 5 mm, matrix = 128 × 128, FOV = 300 mm, and a single average. To reduce the impact of low‐SNR data, a mask with a threshold set to one‐tenth of the maximum B_1_^+^ value was applied to the B_1_^+^ map.

## 4 RESULTS

**FIGURE 5:**
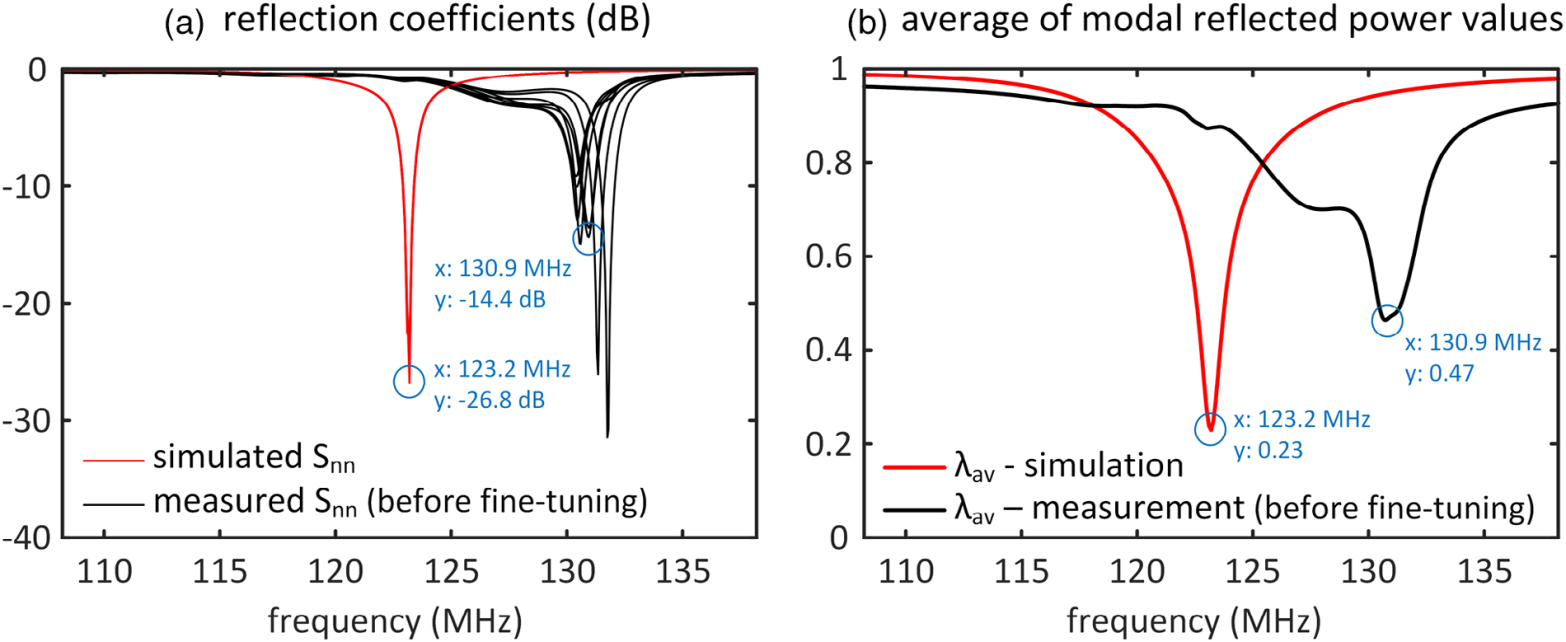
Simulated and measured (a) reflection coefficients and (b) the average of modal reflected power values (*λ_av_*) of the eight‐channel array as a function of frequency. The measurements were done before any fine‐tuning procedure. The same capacitors were used in both simulated and fabricated coils (*c_d_* = 12.8 pF, *c_t_* = 9.5 pF, *c_m_* = 34.7 pF, and *c_s_* = 10.5 pF); however, the capacitors used in the fabricated coil had a ± 5% tolerance.

For the simulated array, capacitor values optimized by the *λ*‐opt approach were 12.8, 9.5, 34.7, and 10.5 pF for *c_d_*, *c_t_*, *c_m_*, and *c_s_*, respectively. The identical capacitor values with a ± 5% tolerance were implemented in the fabricated array. Figure 5 shows the reflection coefficients (|S_nn_|) and the average of modal reflected power values (*λ_av_*) for both simulated and fabricated arrays across frequencies. Note that *λ_av_* is equal to $\frac{1}{8}\sum_{n=1}^{8}\sum_{m=1}^{8}|S_{nm}{|}^{2}$.^33^ The results indicated that all transmit channels of the simulated coil resonate at 123.2 MHz with reflection coefficients below −23 dB, showing excellent tuning and matching. In contrast, the fabricated coil's transmit channels resonate at different frequencies (130.4–131.8 MHz) with varying matching levels (‐9  to −31 dB). On average, the resonances are 7.7 MHz higher than the desired resonance frequency. Figure 5b reveals that the lowest *λ_av_* for the simulated array is 0.23 at 123.2 MHz, whereas for the fabricated array, the lowest *λ_av_* is 0.47 at 130.9 MHz. Since *λ_av_* is also equal to $\frac{1}{8}\sum_{n=1}^{8}\sum_{m=1}^{8}|s_{\mathrm{nm}}{|}^{2}$,^33^ it suggests differences in other **S**‐parameters between the simulated and fabricated arrays.

Supporting Information Figure S1 shows the *λ_n_* values plus *λ_CP_* of the simulated array at 123.2 MHz and the fabricated array at 123.2  and 130.9 MHz. The measured *λ* values are consistently higher than the simulated values. For the fabricated coil at its resonance frequency (130.9 MHz), only five eigenmodes, that is, the eigenvector of ***S*** *^H^* ***S***, have a total reflection of ≤ 50%, while at the desired frequency (123.2 MHz), all eigenmodes of the fabricated coil exhibit a total power reflection of ≥ 50%.

**FIGURE 6:**
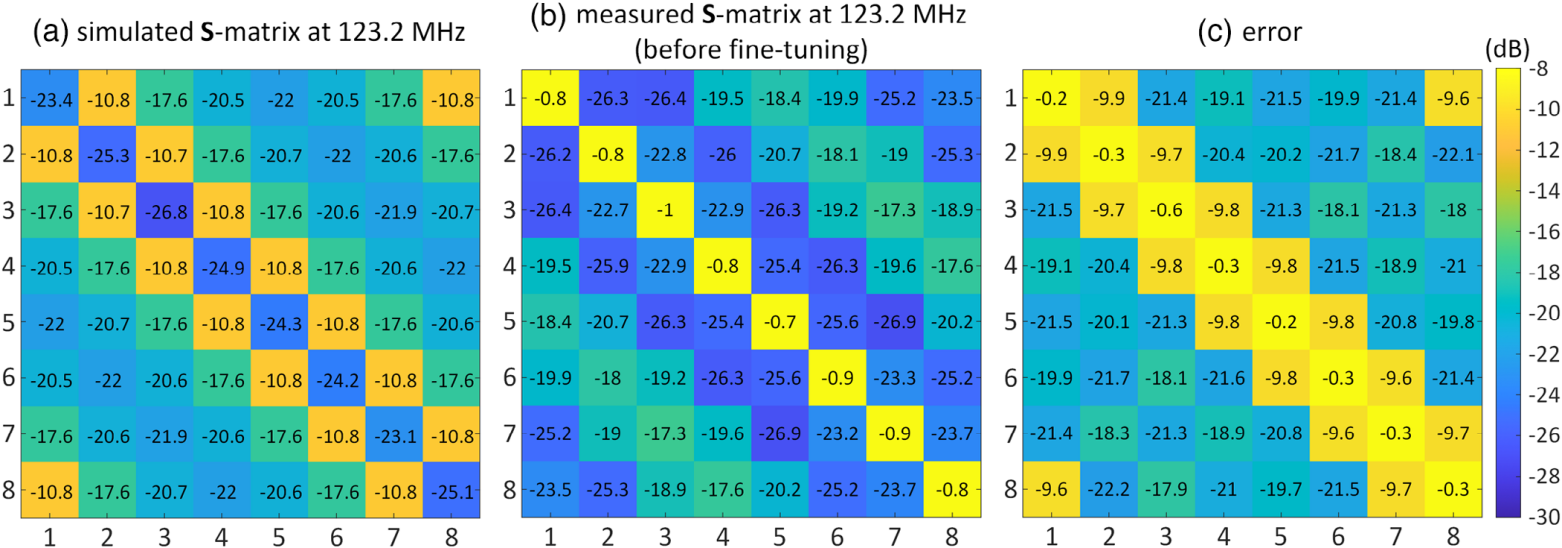
(a) Simulated and (b) measured **S**‐matrices both at 123.2 MHz of the eight‐channel array. (c) Difference between the simulated and measured **S**‐matrices. The **S**‐measurements of the fabricated coil were done before any fine‐tuning procedure.

Figure 6a,b present the simulated and measured **S**‐matrices at 123.2 MHz for the eight‐channel array. An error matrix, defined as the absolute difference between the simulated and measured **S**‐matrices (*|* ***S*** *_simulated_—* ***S*** *_measured_|*) at 123 MHz, is illustrated in Figure 6c. Figure S2 presents the measured **S**‐matrix of the faricated coil at its resonance frequency (130.9 MHz) and its difference from the simulated **S**‐matrix at 123.2 MHz, calculated as *|* ***S*** *_simulated_ (f = 123.2 MHz)—* ***S*** *_measured_ (f = 130.9 *MHz)*|*. In response to the observed disparities between simulated and measured results when employing identical capacitors in both fabricated and simulated arrays, we attribute these differences to inherent imperfections in the coil's construction. This suggests that the fabricated and simulated structures are not perfectly identical. To address this mismatch, we applied the proposed fast fine‐tuning approach to the fabricated array, involving measurements of its inductance and resistance matrices. Subsequently, new capacitor values were determined through fine‐tuning based on the λ‐opt approach. Specifically, the refined capacitor values for *c_d_*, *c_t_*, *c_m_*, and *c_s_* were determined as 14.5 ± 0.1, 10.6 ± 0.7, 40.4 ± 0.2, and 12.9 ± 0.1 pF, respectively.

**FIGURE 7:**
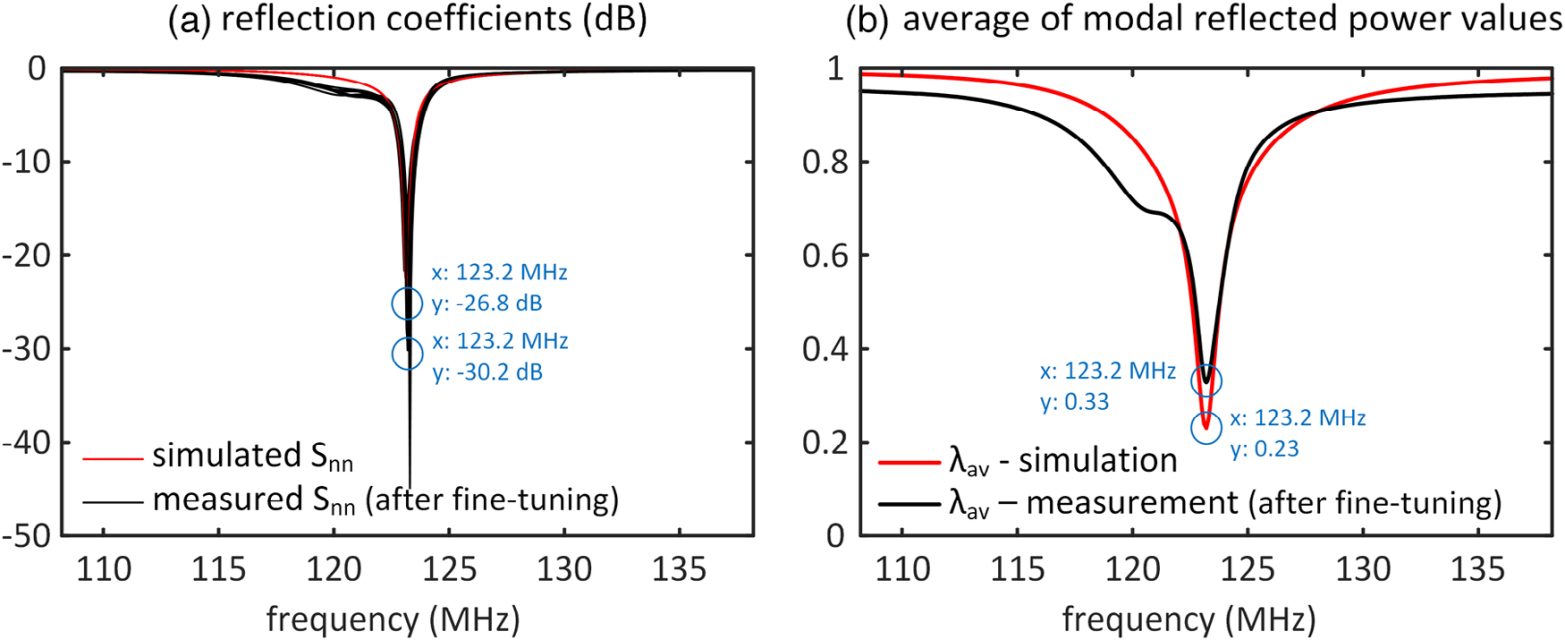
Simulated and measured (a) reflection coefficients and (b) average of modal reflected power values (*λ_av_*) for the eight‐channel array as a function of frequency. Simulation results were obtained with the initial capacitor values (*c_d_* = 12.8 pF, *c_t_* = 9.5 pF, *c_m_* = 34.7 pF, and *c_s_* = 10.5 pF), while the measurement results were achieved with the fine‐tuned capacitor values (*c_d_* = 14.5 ± 0.1 pF, *c_t_* = 10.6 ± 0.7 pF, *c_m_* = 40.4 ± 0.2 pF, and *c_s_* = 12.9 ± 0.1 pF).

Figure 7 illustrates the reflection coefficients and *λ_av_* for the fabricated array with the updated set of capacitors as a function of frequency. The reflection coefficients and *λ_av_* for the simulated array are also presented for comparison. In Figure 7a, it is evident that all transmit channels of the fabricated coil resonate at the desired resonance frequency with an acceptable matching level (≤ −15 dB). Figure 7b further demonstrates that the lowest value of *λ_av_* for the fabricated coil is 0.33, occurring at 123.2 MHz. While this value is greater than the corresponding simulation, it remains the lowest achievable *λ_av_* for the fabricated array.

**FIGURE 8:**
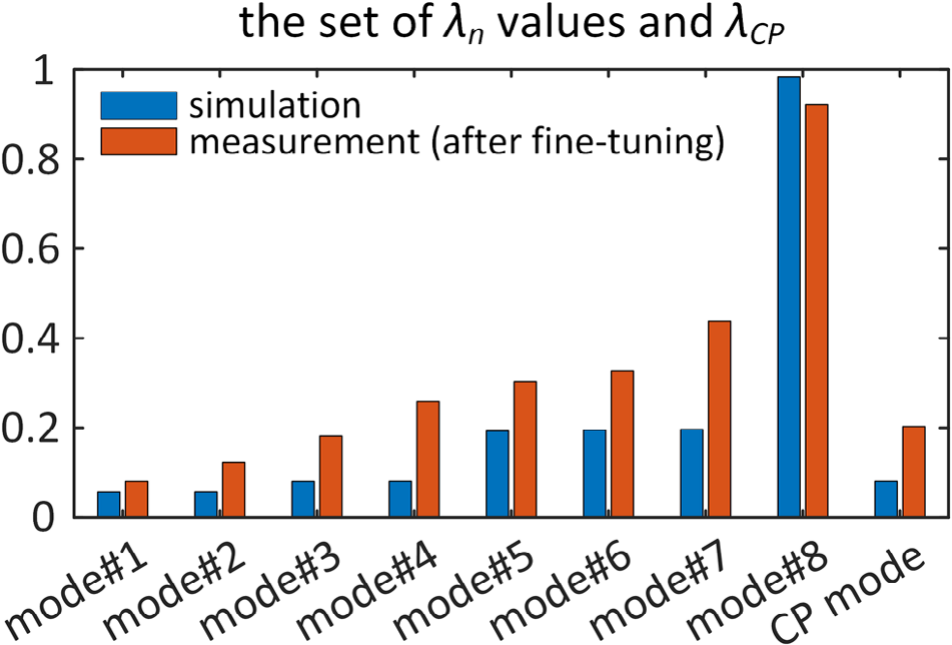
Simulated and measured modal reflected power values for the eight‐channel array at 123.2 MHz. Additionally, *λ_CP_* is included for comparison. The simulated *λ* values were obtained using the initial capacitor settings, while the measured *λ* values were obtained with the fine‐tuned capacitor values.

In Figure 8, the measured *λ* values at 123.2 MHz for the fine‐tuned fabricated array are presented alongside the simulated *λ* values. Both simulated and fabricated coils exhibit only one excitation eigenmode with an extremely high modal reflected power (> 0.92), indicating minimal contribution to the transmission process. The simulated coil displays seven excitation eigenmodes with a total reflection of less than 20%, whereas the fabricated coil has seven excitation eigenmodes with a maximum total reflection of less than 50%. The normalized reflected power in the CP excitation mode for the simulated and fabricated coils is 0.08 and 0.2, respectively. In the fine‐tuning process of the fabricated coil, there was a reduction in *λ_CP_*, though it led to an increase in *λ_av_*.

**FIGURE 9:**
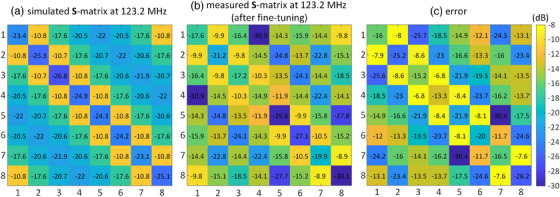
(a) Simulated and (b) measured **S**‐matrices of the eight‐channel array at 123.2 MHz. (c) The difference between the simulated and measured **S**‐matrices at 123.2 MHz. Simulated **S**‐matrix was obtained with the initial capacitor values, while the measured **S**‐matrix was achieved with the fine‐tuned capacitor values.

Figures 9a,b present the **S**‐matrices of the simulated and fine‐tuned fabricated arrays at 123.2 MHz. The maximum coupling between neighboring channels is −10.7 dB for the simulated array and −8.9 dB for the fabricated array. For non‐neighboring channels, the maximum coupling is −17.6 and −13.5 dB for the simulated and fabricated arrays, respectively. Additionally, Figure 9c shows the error matrix, defined as the absolute difference between the simulated and measured **S**‐matrices at 123.2 MHz. The maximum difference in matching and coupling levels are −13.3  and −6.8 dB, respectively, which is lower than the error presented in Figure 6c. It is important to note that in the fine‐tuning process, only the cost function specified in Equation (1) at 123.2 MHz was minimized, and no attempt was made to reduce the difference between the simulated and measured **S**‐matrices.

**FIGURE 10:**
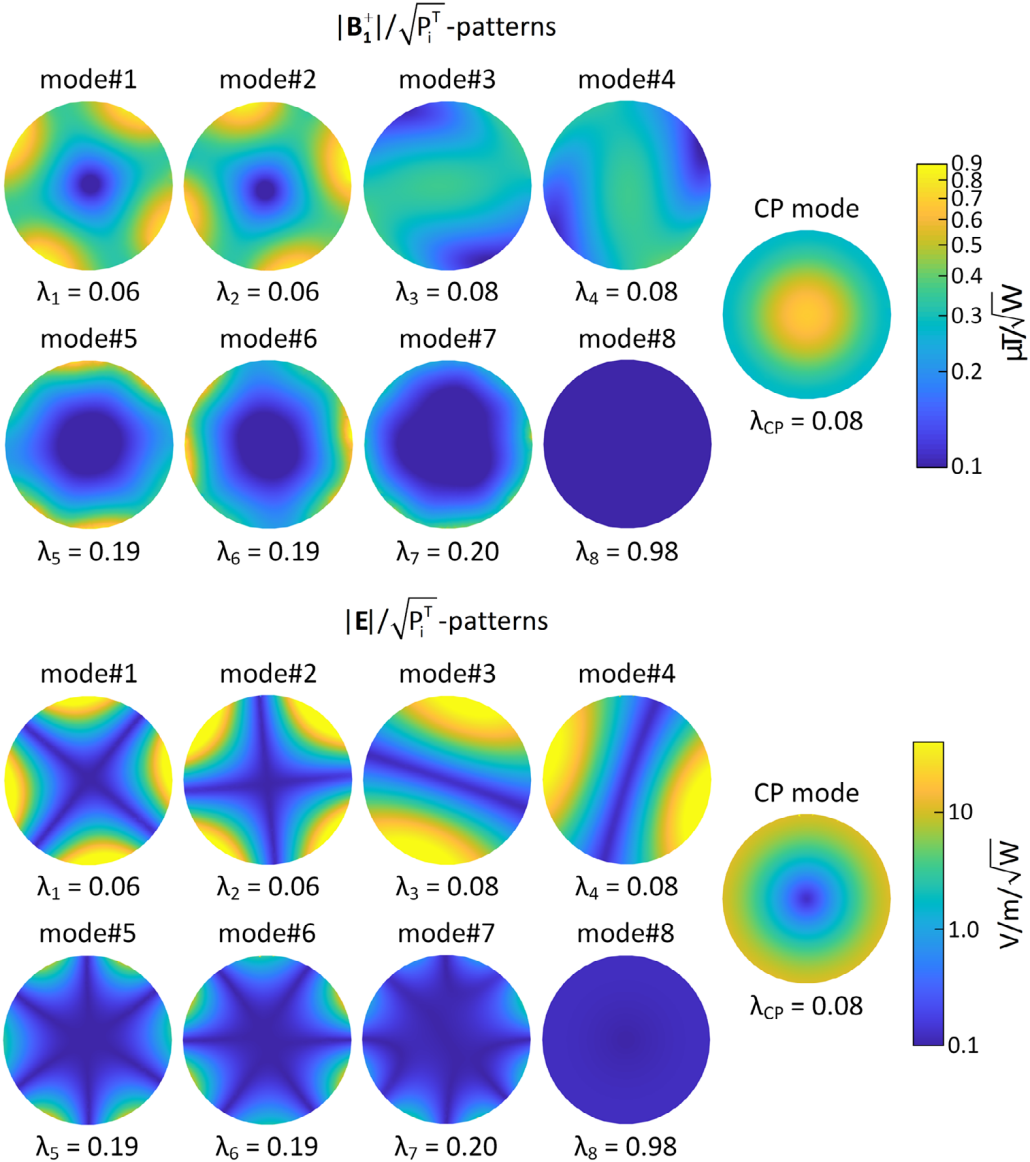
B_1_^+^ and E‐field patterns of the simulated eight‐channel array with initial capacitor values. Patterns are shown at the central axial plane at 123.2 MHz for all eight eigenmodes and CP excitation, normalized by the square root of the total incident power.

Figure 10 depicts simulated B_1_^+^ and electric field patterns of all eight eigenmodes of the eight‐channel array, sorted by total reflection. The CP execution mode field patterns are also shown. All patterns are at 123.2 MHz within the phantom at the central axial plane and normalized by the square root of the coil's total incident power. The CP excitation vector expansion reveals that CP fields are primarily a combination of fields generated by the 3rd and 4th excitation eigenmodes. These two modes produce linearly‐polarized B_1_^+^ fields orthogonal to each other. The CP mode B_1_^+^ efficiency, calculated as the average normalized B_1_^+^ within the phantom at the central axial plane, is 0.57 µT/$\sqrt{W}$. Supporting Information Figure S3 also presents a comparison of the B_1_^+^ patterns between the fabricated and simulated eight‐channel arrays at the central axial plane, with both coils operating in CP mode.

## 5 DISCUSSION

The results presented in this study highlight the challenges associated with fine‐tuning transmit arrays, particularly when aiming for optimal performance in MRI applications. The accelerated fine‐tuning methodology introduced in this study leverages equivalent circuit modeling and eigenmode analysis, presenting a practical and efficient approach to refining the performance of transmit arrays. The integration of an equivalent circuit model with measured **S**‐parameters allows for a rapid adjustment of capacitor values, minimizing mismatches between simulation and experimental outcomes. This approach addresses the inherent complexities of fine‐tuning, especially in the case of multi‐channel arrays, by streamlining the iterative process. The successful application of this methodology to an eight‐channel degenerate birdcage coil underscores its potential for enhancing the efficiency of the array design process.

The discrepancies observed between the simulated and fabricated arrays, as depicted in Figures 5 and 6, underscore the intricacies introduced during the manual fabrication process. When implementing capacitors directly obtained from simulations into the fabricated coil, a notable mismatch in performance becomes evident. This discrepancy can be attributed to several interconnected sources, the primary one being the physical mismatch introduced during manual fabrication. The manual construction process introduces imperfections, including variations in the sizes of copper segments distributed along the axial direction, non‐parallel rungs, and non‐uniform spacing among segments. These factors collectively contribute to the observed disparities in resonance frequencies and matching levels. Additionally, the coil and shield structures, situated on plexiglass frames lacking sufficient strength and stability, deviate from perfect co‐centering along the axial direction. These imperfections in the physical construction inherently lead to deviations from the ideal simulated model, emphasizing the challenges associated with achieving precise alignment and uniformity in laboratory‐fabricated transmit arrays. Addressing these physical mismatches becomes imperative for aligning the fabricated array with simulation expectations. The proposed accelerated fine‐tuning methodology, incorporating equivalent circuit modeling and eigenmode analysis, effectively mitigates these disparities. By iteratively adjusting capacitor values based on the measured inductance and resistance matrices, the methodology compensates for the imperfections introduced during fabrication, aligning the fabricated array more closely with the simulated ideal.

The proposed accelerated fine‐tuning methodology introduces a notable departure from conventional approaches, demonstrating remarkable efficiency by achieving optimal capacitor values with a limited number of iterations. In the case of the tested coil, a single iteration was sufficient to refine the capacitor values. Conventionally, the fine‐tuning of Tx arrays involved adjusting capacitors individually, often relying on the iterative process of changing one element at a time and assessing its impact on the overall performance. This traditional approach not only demands a significant time investment but also necessitates a keen understanding of the intricate interactions between array elements. Our proposed fast‐fine tuning approach offers a substantial departure from this conventional methodology. By leveraging equivalent circuit modeling and measured **S**‐parameters, we streamline the fine‐tuning process. The methodology allows for a more comprehensive adjustment of capacitor values, addressing the collective behavior of the array elements rather than relying on individual adjustments.

In this study, we employed our recently developed array design method, which relies on eigenmode analysis of the **S**‐matrix,^33^ to determine optimal capacitor values. Eigenmode analysis of the **S**‐matrix provides an efficient means of quantitatively representing the power transfer capabilities of Tx arrays. This analysis categorizes excitation modes of a pre‐designed coil based on their power reflection levels. It has been demonstrated that the normalized reflected power for a specific excitation mode can be calculated as the weighted sum of the eigenvalues of the **S** ^H^ **S**‐matrix (H represents the Hermitian transpose).^33^ Minimizing the eigenvalues of the **S** ^H^ **S**‐matrix expands the excitation space with low power reflection. The eigenmode analysis serves as a valuable tool for evaluating, comparing, and optimizing the transmission performance of Tx arrays. In this study, we applied this approach to optimize the coil's capacitor values, aiming to minimize all eigenvalues of the **S** ^H^ **S**‐matrix and the normalized reflected power in CP excitation.

In simulating the eight‐channel tested array, we performed a full‐wave EM field simulation, coupled with a co‐simulation strategy.^32^ In the co‐simulation method, all capacitors were substituted with lumped ports. Although this approach demands more time and computational resources, it offers flexibility for rapid fine‐tuning, matching, and decoupling of the array within a circuit simulator. Despite its apparent complexity and time‐consuming nature, the co‐simulation method could be employed in the design of the fabricated coil. This involves measuring the **S**‐parameters of the fabricated array by replacing all capacitors with ports, mirroring the approach used in the simulation environment.

The implementation of the co‐simulation method for the eight‐channel fabricated coil required treating it as a 40‐port system (8 real ports + 32 capacitors), necessitating 820 individual **S**‐measurements using a two‐port network analyzer. Additionally, all capacitors had to be substituted with coaxial cables equipped with cable traps to accurately measure the 40‐port **S**‐parameters. However, the introduction of extra cabling near the transmit loops can influence the coil's EM fields, introducing errors into the measured **S**‐parameters. Furthermore, the additional coaxial cables introduce extra phases to the measured **S**‐parameters. These additional phases must be precisely measured and subtracted from the 40‐port **S**‐parameters during post‐processing. As the number of Tx channels and capacitors increases, these issues become more pronounced, rendering this method inefficient. In contrast, our proposed approach presents a streamlined version of the aforementioned co‐simulation method for the fabricated array. This method eliminates the necessity of replacing capacitors with ports, preserving accuracy and diminishing the need for numerous **S**‐measurements. While the co‐simulation method demands knowledge of the **S**‐parameters of the 40‐port system, our approach suggests measuring **S**‐parameters of the 8‐port system terminated with known capacitors and analyzing the equivalent circuit model of the 40‐port system to estimate the inductance and resistance matrices of the eight‐port system. This estimation facilitates faster fine‐tuning of the capacitors.

While the fine‐tuning approach presented in this study could be applied without EM simulations, simulations play an essential role in ensuring the effectiveness of the overall process, especially for complex arrays with a higher number of transmit channels. For simpler designs, where finding an initial guess for capacitors is easier, direct **S**‐parameter measurements and manual fine‐tuning may suffice. However, as design complexity increases, EM simulations help provide an initial set of capacitor values, reducing the number of fine‐tuning iterations needed by offering a reliable, partially optimized starting point.

## 6 CONCLUSION

This study presents a practical and efficient methodology for the accelerated fine‐tuning of RF transmit arrays, using an eight‐channel degenerate birdcage coil designed for 3T MRI as a case study. Acknowledging the intricate challenges associated with fine‐tuning transmit arrays, especially concerning increased coupling complexities, we introduce an approach that combines equivalent circuit modeling and eigenmode analysis. Our methodology involves integrating the measured **S**‐parameters of a fabricated coil, utilizing identical capacitors as the simulated coil, into an equivalent circuit model. By calculating the inductances and resistances of the coil's circuit model and subsequently adjusting capacitor values, we achieved rapid and effective fine‐tuning. This approach proved instrumental in mitigating disparities between simulated and experimental results, a critical consideration in the context of lab‐fabricated arrays. Our case study with the eight‐channel coil demonstrated marked improvements in resonance and matching characteristics, along with a substantial reduction in power reflected from the coil.

## CONFLICT OF INTEREST STATEMENT

The authors have no relevant conflicts of interest to disclose.

## Supporting information



Supporting Information

## APPENDIX A

### A.1

The **S**‐matrix is represented as the relationship between the incident and reflected moving waves (Supporting Information Figure S4), and it can be defined as **b **=** Sa**, where **b** and **a** are, respectively, the vectors of the reflected and incident power waves, described as the following linear transportations

(A1)
$$\begin{array}{ccc} b=\frac{1}{2\sqrt{\left|ℜ\{Z_{0}\}\right|}}\left(V-Z_{0}^{*}I\right) & \mathrm{and} & a=\frac{1}{2\sqrt{\left|ℜ\{Z_{0}\}\right|}}\left(V+Z_{0}I\right) \end{array}$$

where *Z_0_* denotes the coil's reference impedance, and in this study is chosen as 50 ohms. **V** and **I** are the vectors of the total voltage and current. The total average power transmitted to the Tx array system can be calculated as the difference between the total incident power and the total reflected power^62^; therefore, it can be expressed as

(A2)
$$P_{t}^{T}=P_{i}^{T}-P_{r}^{T}=a^{H}a-b^{H}b=a^{H}\left(U-S^{H}S\right)a$$

where the superscript **H** represents the Hermitian transpose operation, and **U** represents the identity matrix. The reduction of the total reflected power would increase the net power transmitted to the Tx array for a fixed amount of total incident power. As a result, the ratio of the total reflected power to the total incident power, called the *normalized reflected power*, should be minimized to increase the array's power efficiency. For a specific vector of incident power waves, the normalized reflected power can be written as

(A3)
$$\lambda \left(a\right)=\frac{P_{r}^{T}}{P_{i}^{T}}=\frac{b^{H}b}{a^{H}a}=\frac{a^{H}S^{H}\mathrm{Sa}}{a^{H}a}$$

The form of Equation (A3) is a Rayleigh quotient,^63^ which has the property that

(A4)
$$\lambda_{\min}\leq \lambda \left(a\right)\leq \lambda_{\max},\;\;\;\;\;\;\mathrm{for}\;\mathrm{all}\;\;a\in C^{N}$$

where *λ_min_* and *λ_max_* denote the smallest and largest eigenvalues of **S^H^S**‐matrix, respectively. Note that **S^H^S**‐matrix is a Hermitian matrix, and its eigenvalues are always positive real numbers with values less than or equal to one.^64^ This matrix can be diagonalized by a unitary similarity transformation^65^ as follows

(A5)
$$S^{H}S=Q\Lambda Q^{H}$$

where Λ is a diagonal matrix composed of the eigenvalues of **S^H^S**‐matrix, and **Q** is a unitary matrix (i.e., **Q^H^Q = QQ^H^ = U**) whose columns are the eigenvectors of **S^H^S**‐matrix. For a generic Tx array, assume that the scalar *λ_n_* and the nonzero vector $q_{n}$ are the *n*th eigenvalue and *n*th eigenvector of the corresponding **S^H^S**‐matrix, respectively, which satisfy $S^{H}Sq_{n}=\lambda_{n}q_{n}$. Consider the condition in which the array is excited so that the vector of incident voltage waves is the same as the *n*th eigenvector of **S^H^S**‐matrix, that is, $a=q_{n}$, then the normalized reflected power can be found as

(A6)
$$\lambda \left(a=q_{n}\right)=\frac{q_{n}^{H}S^{H}Sq_{n}}{q_{n}^{H}q_{n}}=\frac{q_{n}^{H}\lambda_{n}q_{n}}{q_{n}^{H}q_{n}}=\lambda_{n}$$

The eigenvectors of **S^H^S**‐matrix are linearly independent^64^ where they can span the excitation space of the Tx array. They can be considered as unique basis vectors and can be called the *excitation eigenmodes* of the Tx array coil. Consequently, the eigenvalues of the **S^H^S**‐matrix can be called the *modal reflected power* values. In general, each vector of incident power waves can be expanded as a sum of different excitation eigenmodes, that is, $a=\sum_{n=1}^{N}w_{n}q_{n}=\mathrm{Qw}$, where $w={\left[\begin{array}{cccc} w_{1} & w_{2} & \text{…} & w_{N} \end{array}\right]}^{T}$ is composed of the expansion coefficients; therefore, the normalized reflected power can be written as

(A7)
$$\begin{array}{cc} \lambda (a) & =\frac{a^{H}S^{H}\mathrm{Sa}}{a^{H}a}=\frac{w^{H}Q^{H}S^{H}\mathrm{SQw}}{w^{H}Q^{H}\mathrm{Qw}}=\frac{w^{H}Q^{H}Q\Lambda Q^{H}\mathrm{Qw}}{w^{H}Q^{H}\mathrm{Qw}}\\ & =\frac{w^{H}\Lambda w}{w^{H}w}=\sum_{n=1}^{N}\left(\lambda_{n}{\left(\frac{w_{n}}{\left\|w\right\|}\right)}^{2}\right) \end{array}$$

Equation (A7) demonstrates the normalized reflected power for a given vector of incident power waves as a normalized sum of the modal reflected power values. To design Tx arrays in a power‐efficient manner, the design parameters which are effective in the determination of the **S^H^S**‐matrix can be optimized to minimize its eigenvalues, thereby ensuring the total reflected power is low for a broader set of incident power waves.

## APPENDIX B

### B.1

Assuming a zero current for the mesh formed by the lower end‐ring and employing the Kirchhoff mesh current method to analyze the circuit model of the fabricated array depicted in Figure 4, the following matrix equation is derived:

(B1)
$$\left(j\omega \;L\left(\omega \right)+\frac{1}{j\omega }C+R\left(\omega \right)\right)i\left(\omega \right)=\frac{1}{j\omega }C_{m}I\left(\omega \right)$$

where ω is the angular frequency and $j=\sqrt{-1}$. $i\left(\omega \right)={\left[i_{q}\left(\omega \right)\right]}_{8\times 1}$ denotes the vector of loop currents and $I\left(\omega \right)={\left[I_{q}\left(\omega \right)\right]}_{8\times 1}$ denotes the vector of current sources. $C={\left[C_{q,p}\right]}_{8\times 8}$ and $C_{m}={\left[C_{q,p}^{m}\right]}_{8\times 8}$ represent the capacitance matrices, where $C_{q,p}$ and $C_{q,p}^{m}$ are defined as

(B2)
$$C_{q,p}=\left\{\begin{array}{cc} \frac{1}{c_{d}^{q-1}}+\frac{1}{c_{d}^{q}}+\frac{1}{c_{t}^{q}}+\frac{1}{c_{m}^{q}} & \mathrm{if}\;q=p\\ -\frac{1}{c_{d}^{\min\{q,\;p\}}} & \mathrm{if}\;q-p\equiv \pm 1\;\;(\mathrm{mode}8)\\ 0 & \mathrm{otherwise} \end{array}\right.$$

(B3)
$$C_{q,p}^{m}=\left\{\begin{array}{cc} \frac{1}{c_{m}^{q}} & \mathrm{if}q=p\\ 0 & \mathrm{otherwise} \end{array}\right.$$

$L={\left[L_{q,p}\right]}_{8\times 8}$ and $R={\left[R_{q,p}\right]}_{8\times 8}$ also represent the inductance and resistance matrices, respectively, in which $L_{q,p}$ and $R_{q,p}$ are given by

(B4)

(B5)

Following the Kirchhoff mesh current method, the *q*th voltage source can be determined as

(B6)
$$V_{q}\left(\omega \right)=\frac{1}{j\omega }\left(\frac{1}{c_{m}^{q}}+\frac{2}{c_{s}^{q}}\right)I_{q}\left(\omega \right)-\frac{1}{j\omega c_{m}^{q}}i_{q}\left(\omega \right)$$

This equation can be expressed in matrix form as

(B7)
$$V\left(\omega \right)=\frac{1}{j\omega }\left(C_{m}+2C_{s}\right)I\left(\omega \right)-\frac{1}{j\omega }C_{m}i\left(\omega \right)$$

where $V\left(\omega \right)={\left[V_{q}\left(\omega \right)\right]}_{8\times 1}$ denotes the vector of voltage sources and $C_{S}={\left[C_{q,p}^{s}\right]}_{8\times 8}$, with elements given by

(B8)
$$C_{q,p}^{s}=\left\{\begin{array}{cc} \frac{1}{c_{s}^{q}} & \mathrm{if}q=p\\ 0 & \mathrm{otherwise} \end{array}\right.$$

Substituting the vector of loop input currents obtained from Equation (B1) into Equation (B7) gives

(B9)
$$\begin{array}{cc} V(\omega ) & =\left(\frac{1}{j\omega }\left(C_{m}+2C_{s}\right)\right.\\ & \;+\left.\frac{1}{\omega^{2}}C_{m}{\left(j\omega \;L\left(\omega \right)+\frac{1}{j\omega }C+R\left(\omega \right)\right)}^{-1}C_{m}\right)I\left(\omega \right) \end{array}$$

Therefore, the impedance (**Z**) matrix of the fabricated coil's model can be expressed as follows:

(B10)
$$\begin{array}{cc} Z(\omega ) & =\frac{1}{j\omega }\left(C_{m}+2C_{s}\right)\\ & \;+\frac{1}{\omega^{2}}C_{m}{\left(j\omega \;L\left(\omega \right)+\frac{1}{j\omega }C+R\left(\omega \right)\right)}^{-1}C_{m} \end{array}$$

The **Z**‐matrix can then be easily converted to the **S**‐matrix as follows

(B11)
$$S\left(\omega \right)={\left(Z\left(\omega \right)+Z_{0}U\right)}^{-1}\left(Z\left(\omega \right)-Z_{0}U\right)$$

when the optimized capacitor values achieved from the simulation environment are implemented in the the fabricated coil, measuring its **Z**‐parameters over a wide range of frequencies enables the calculation of the inductance and resistance matrices as

(B12)
R(ω)=1ω2ReCmZ(ω)−1jω(Cm+2Cs)−1Cm

(B13)
L(ω)=1ω3ImCmZ(ω)−1jω(Cm+2Cs)−1Cm+1ω2C
