# Supplementary material for: Enhancing fine‐tuning efficiency and design optimization of an eight‐channel 3T transmit array via equivalent circuit modeling and Eigenmode analysis
Source: Med Phys. 2025 Jan 15;52(4):2025–39. doi: 10.1002/mp.17612 (PMC11972053; doi:10.1002/mp.17612)
Supplement: Supplementary file 1 — Supporting Information [file MP-52-2025-s001.pdf]

Enhancing Fine-Tuning Efficiency and Design Optimization of an 8-Channel 3T Transmit Array via Equivalent Circuit Modeling and Eigenmode Analysis

Authors: Ehsan Kazemivalipour[1,2,3,4] and Ergin Atalar[1,2]

[1]Department of Electrical and Electronics Engineering, Bilkent University, Ankara, Turkey
[2]National Magnetic Resonance Research Center (UMRAM), Bilkent University, Ankara, Turkey
[3]A. A. Martinos Center for Biomedical Imaging, Department of Radiology, Massachusetts General Hospital, Charlestown, Massachusetts, USA
[4]Harvard Medical School, Boston, Massachusetts, USA

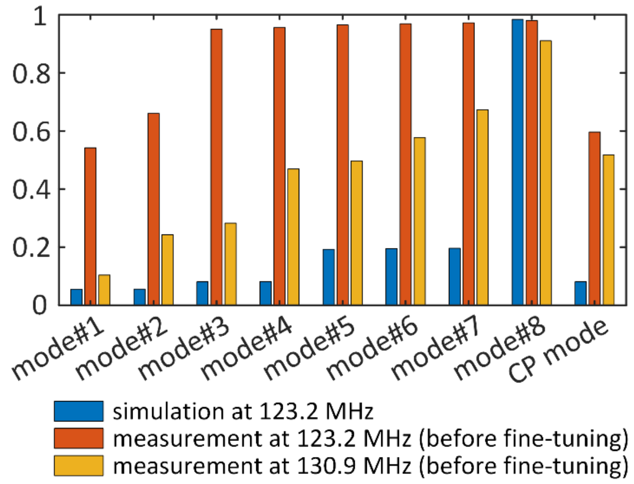

Figure S1 - Modal reflected power values of the simulated 8-channel array at 123.2 MHz and the fabricated 8-channel array at 123.2 MHz and 130.9 MHz. The λ-values measured for the fabricated coil were all calculated prior to any fine-tuning procedure. The graph includes a reference to the modal reflected power value at the circularly polarized mode (λ_{CP}) for comparison. Initial capacitor values (c_d = 12.8 pF, c_t = 9.5 pF, c_m = 34.7 pF, and c_s = 10.5 pF) were consistent for both simulated and fabricated coils, with the fabricated coil's capacitors having a ±5% tolerance.

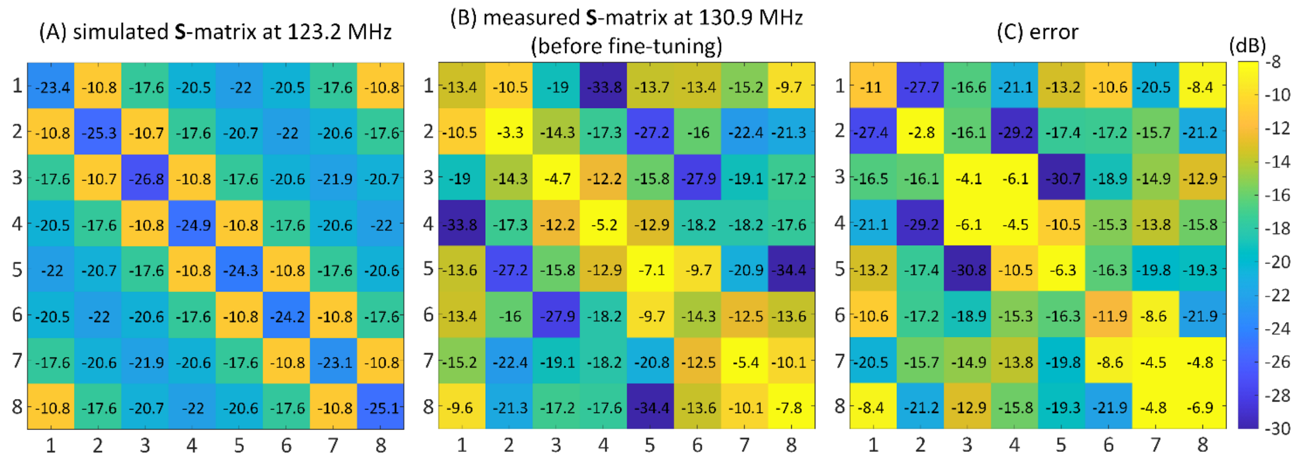

Figure S2 - (A) Simulated **S**-matrix at 123.2 MHz and (B) measured **S**-matrix at 130.9 MHz of the 8-channel array. (C) Difference between the simulated **S**-matrix at 123.2 MHz and measured **S**-matrix at 130.9 MHz. The **S**-measurements of the fabricated coil were done before any fine-tuning procedure.

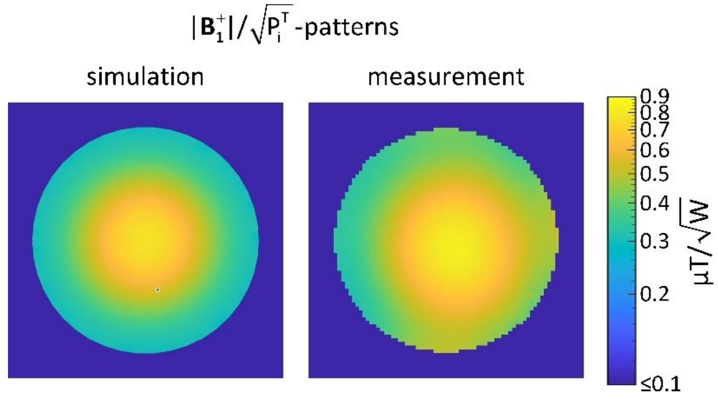

Figure S3 – Simulated and measured $\mathbf{B}_1^+$ patterns of CP excitation mode at the central axial slice of the simulated and fabricated 8-channel Tx array. Field patterns are normalized by the square root of total incident power.

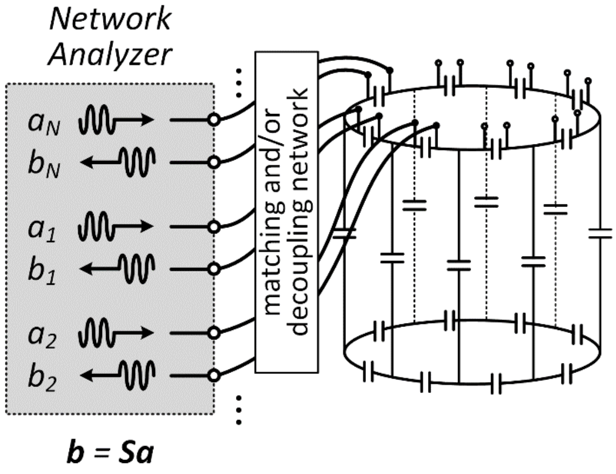

Figure S4 - Schematic representation of the incident and reflected power wave measurement in a Tx array system. The incident power wave (**a**) is introduced to the coil, and the reflected power wave (**b**) is measured using an **S**-parameter Network Analyzer. The measurement is conducted with a reference impedance of Z_0 = 50 Ω.
